# Metamorphosis in the Porosity of Recycled Concretes Through the Use of a Recycled Polyethylene Terephthalate (PET) Additive. Correlations between the Porous Network and Concrete Properties

**DOI:** 10.3390/ma10020176

**Published:** 2017-02-14

**Authors:** José Miguel Mendivil-Escalante, José Manuel Gómez-Soberón, Jorge Luis Almaral-Sánchez, Francisca Guadalupe Cabrera-Covarrubias

**Affiliations:** 1 Faculty of Engineering Mochis, Autonomous University of Sinaloa, Fuente de Poseidón y Ángel Flores s/n, Col. Jiquilpan, Module B2, Los Mochis, Sinaloa 81210, Mexico; josemiguelmendivil@hotmail.com (J.M.M.-E.); jalmaral@uas.edu.mx (J.L.A.-S.); 2 Barcelona School of Building Construction, Polytechnic University of Catalonia, Av. Doctor Marañón 44-50, Barcelona 08028, Spain; 3 Barcelona School of Civil Engineering, Polytechnic University of Catalonia, C. Jordi Girona 1-3, Building C2, Barcelona 08034, Spain; guadalupe.cabrera04@gmail.com

**Keywords:** PET additives, recycled concrete, concrete porosity, polymeric resins, porosimetry of gas adsorption, acoustic resonance spectroscopy

## Abstract

In the field of construction, sustainable building materials are currently undergoing a process of technological development. This study aims to contribute to understanding the behavior of the fundamental properties of concretes prepared with recycled coarse aggregates that incorporate a polyethylene terephthalate (PET)-based additive in their matrix (produced by synthesis and glycolysis of recycled PET bottles) in an attempt to reduce their high porosity. Techniques to measure the gas adsorption, water porosity, Fourier transform infrared spectroscopy (FTIR) and X-ray diffraction (XRD) were used to evaluate the effect of the additive on the physical, mechanical and microstructural properties of these concretes. Porosity reductions of up to 30.60% are achieved with the addition of 1%, 3%, 4%, 5%, 7% and 9% of the additive, defining a new state in the behavioral model of the additive (the overdosage point) in the concrete matrix; in addition, the porous network of these concretes and their correlation with other physical and mechanical properties are also explained.

## 1. Introduction

At its current level, the technological development of concrete is focused on the optimization of its characteristics, components and mechanical properties, particularly in their durability. Parallel to this development, a new sustainable concept for concrete has emerged, known as recycled concrete (RC), which is produced by the mixture of natural stone aggregates and recycled coarse aggregates from demolition waste (in specific of old concrete) (RA_coarse_) agglutinated with a cement paste [1,2]. This development arose as an attempt to satisfy the current need for the use of construction waste [3,4] and the RC has been tested in various countries as a method of conserving natural resources, increasing the useful lifespan of sanitary landfills, and thus avoiding the exploitation of non-renewable natural resources [5].

There are several standards that regulate the use of RA_coarse_ for RC; they have been specified contents from 20% to 50% as the upper limit to use them [6,7,8,9,10,11]. The behavior of the RC in the fresh state, reports a reduction of the workability, causing the need for more water in the mixing [12,13], and of previous saturation of them [14,15]. This performance is related to the composition of the RA_coarse_ [16] (usually 65%–70% of old natural aggregate and 30%–35% of old adhered mortar [17]), which, in turn, responds to its superior absorption capacity (between 5% and 15%) [17,18] and to the low density of RC [19,20] (between 2090 and 2392 kg/m^3^) [14,21,22].

The mechanical behavior of the RC indicates reductions in their compressive strength (between 5.7% and 40%) [21,22,23,24,25,26,27,28], directly related to the increase of their porosity and the creation of weak links in the cement matrix [29]. Similar behavior is reported for the modulus of elasticity (reductions between 4.17% and 45%) [20,30,31], which is explained because considering the RA_coarse_ as the weakest aggregates [20].

Regarding the durability of RC, its high porosity (between 3.9% and 22.6%) [21,32] implies a greater permeability of it, allowing to aggressive agents the possibility to access; therefore the evaluation of it (porosity) allows understanding its durability.

As an alternative for improving the properties of concrete and contributing to environmental sustainability, research is underway into the use of polyethylene terephthalate (PET) as a component of RC. This substance, via its transformation into an unsaturated polyester resin, could be used as an additive to improve the performance of RC [33]. It is frequently found in plastic bottles, which are widely used and are thus the cause of huge quantities of waste [34]. Regarding the technical aspects favoring its application, previous results have shown that this material may reach 80% of its final strength in just one day (interesting for concretes); however, it may also show strength losses if subjected to high temperatures (comparable with conventional concrete) [35]. New and more sustainable applications can be developed in order to reduce this waste, such as new polymer variants and textile applications, or its possible use as a densifier for concretes (new additives) [36].

Generally, the use of polymers to replace fractions of the aggregates in concretes began in the 1960s [37]. In particular, experiments were carried out on the partial substitution of fine aggregate by crushed polymers, obtaining lighter and more ductile concretes with greater resistance to penetration by chloride ions; on the other hand, the workability, compressive strength and resistance to attraction are properties which are weakened by the inclusion of polymers [38]. This can be explained by aspects of the PET’s own resistance, as well as its capacity to bond with the other compounds within the matrix of the concrete. The maximum percentage permitted for the replacement of fine aggregate by PET granules was established as 25%, above which losses in compressive strength and the elasticity module must be expected (5.82% and 10.31% respectively) [39]; in this case it would seem that the beneficial effect of low dosages of PET are outweighed by the damaging effects of high contents.

On the other hand the uses of PET fiber in concrete, either as a reinforcement to improve the flexural and traction behavior or as an indirect improvement in mitigating the process of corrosion, have already been described in several studies [40,41,42]. In particular for the case of flexural strength—the most acceptable general application for obtaining greater benefits in behavior—it was established that polymer fibers bigger than 50 mm improved this property considerably, with the opposite occurring when they are smaller [43]; this can be understood as being due to the grip or binding effect (reduction of fissures) of the compounds that form the matrix towards the PET fiber when the system undergoes this mechanical effect. In the particular case of mortars containing fibers, it was established that their incorporation does not modify the compressive strength behavior, showing that contents of up to 1.5% fiber produce the best results [44]. On the other hand, the use of polypropylene/polyethylene polymeric hybrid fiber (contents below 2.9%) increases the compressive strength by up to 38%; this improvement is related to the roughness and size of the fibers used, which have a reinforcing effect in the matrix [45].

Nowadays, the evolution of the application of polymers in concretes has enabled the creation of a polymer-modified concrete (PMC), comprising conventional concrete mixed with a polymer resin [46], with PET being the polymer used in this case. This substance, transformed into an unsaturated polyester resin, could be used as an additive to improve the performance of RC [33], while also reducing the production costs of polymer concretes and making savings in energy use.

Previous studies indicate that increases in mechanical strength (of between 15% and 20%) may be obtained in PMC, the origin of these improvements lying in the strong bond of the resin itself with the cementing matrix [47]; this has also been validated in other studies where the advantages of its use in terms of good resistance, durability and rapid curing have been clearly shown [48]. PMC is used in structural applications, pavements, wastewater pipes, repairs, concrete surface coatings etc., providing sufficient durability levels in all applications [49,50] with regard to environmental effects, chemical attacks, abrasion, or any other process [51]. On the other hand, the durability of a concrete tends to be linked to the process of water penetration, which transports the aggressive agent and, in turn, depends on the porosity of the concrete. This is a function of the pore structures and their degree of connectivity, being the physical properties that finally establish its structure [52] (capillary absorption, permeability and diffusion).

While it has been shown that the partial or total replacement of natural coarse aggregates by RA_coarse_ to produce an RC has a positive effect on environmental impact [53,54], this also causes an increase in the porosity of the RC produced [21], which has repercussions in terms of durability.

Therefore, this investigation justified to incorporate an additive or resin derived from waste PET bottles, to improve the porosity properties of RC (of high porosity), allowing to produce an increase in the densification of the paste of them, and thus reverse this effect. This work proposes the study of porosity in these concretes as a comparative instrument and as an explanation of their behavior.

## 2. Materials and Methods

### 2.1. Materials

The following processes were used to obtain the PET-based additive [55]. Firstly, the polymeric resin was synthesized from a recycled PET base taken from plastic bottles post-consumption, with the prior elimination of any non-polymer material; their size was then reduced with a scissors-type cutting tool, the crushed material was then washed in a 50% solution of water and caustic soda to guarantee the elimination of impurities. The second phase in obtaining the additive involved a depolymerization (glycolysis) process undertaken in a reactor (Globe Chemical Reactor, Syrris brand, Royston, UK), using propylene glycol (50% of the weight of the PET) as a solvent, and zinc acetate (0.5% of the weight of the PET) as a catalyst. Finally, the process of synthesizing the resin was carried out on the resulting product, which involved adding maleic anhydride and adipic acid at a molar ratio of 1.1:0.5:0.5, with the aim of obtaining the PET additive to be used in the research (recycled resin, RR).

The recycled aggregate is taken from concrete used in hydraulic pavement and recovered from an uncontrolled landfill (original concrete, OC), which was then subject to crushing (Jaw Crusher, Mod. JS-0804, Manek brand, Mumbai, India), and sieved to obtain a suitable size for use as an aggregate for concrete. The basic properties of the OC are shown in Table 1.

**Figure 1:**
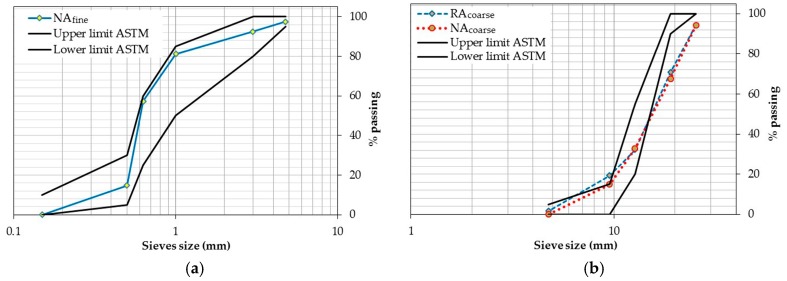
(**a**) Granulometry of natural aggregate fine (NA_fine_); (**b**) Granulometry of coarse aggregates (recycled [RA_coarse_] and natural [NA_coarse_]).

ASTM C136 [56] was used to characterize the distribution of the particle size of the aggregates, obtaining the granulometric profiles from this test for the natural fine aggregates (NA_fine_) (Figure 1a), natural coarse aggregates (NA_coarse_), and RA_coarse_ (Figure 1b). The NA_fine_ used was found to be within the ASTM C33/C33M limits [57], while the profiles of both coarse aggregates used exceeded the lower limit of between 12.7 and 25.4 mm, which indicates the existence of particle content of a larger size than that specified in the regulations. However, this characteristic is common in both aggregates as well as those habitually used in local pre-mixing plants. Therefore, it can be said that partial substitution will not affect the behavior of the concretes containing them, at least not as a result of the effect of their particle distribution.

With regard to the physical properties of the aggregates (ASTM C 642) [58], it should be noted (see Table 2) that the properties of the RA_coarse_ produced are seen to be affected by the singularity of the OC. In particular, the high absorption of the OC causes up to five times more absorption in the RA_coarse_ than in the NA_coarse_ (caused by the old mortar sticking to the natural sand); however, it should be noted that both are in accordance with the regulations. To a lower extent, density reductions of up to 8% [20] are reported for the RA_coarse_ compared to the NA_coarse_. In both cases (absorption and density), the variations detected are attributed to the porous nature of the old mortar sticking to RA_coarse_ [27].

### 2.2. Methods and Experimentation Process

For the purpose of this research it was necessary to use several experimental techniques; standardized tests for determining the physical properties and mechanical trials, as well as more specific and complex techniques and procedures. They are listed in Table 3, below.

The preparation of the specimens of concrete modified with polymer and recycled aggregate (RPC) used a water/cement ratio (w/c) of 0.50% and 25% of NA_coarse_ substituted for RA_coarse_ in the samples, producing six types of mixtures whose sole modification was the quantity of RR content used (% added depending on the weight of the cement). Furthermore, as a control variable, one more mixture was prepared without the addition of RR (AF-0; where AF = Addition Factor of RR, and 0 = zero percent RR addition). The values used for the dosages are shown in Table 4.

The mixing process involved introducing the components into the mixer in the following order: the cement content and the NA_fine_ (in a dry state) were put into the mixer for one minute at mixing speed; the RA_coarse_ and NA_coarse_ were then added to the mixture (both in a saturated surface-dry condition, achieved by immersion in water for 24 h, and after evaporation of the excess by extended and ventilation); this was left in the moving mixer for one minute more until homogenization occurred. Water was then added to the mixture, which was then left to stand for 30 s in order to saturate the NA_fine_ and begin hydrating the cement; finally, the mixing process was carried out for one minute, with an extra minute being added in order to fully incorporate the RR into the mixture.

Six cubic specimens (10 × 10 × 10 cm) (three for compressive strength and three more for apparent density, pore volume and water absorption) and three prismatic specimens (16 × 16 × 60 cm) (for module of elasticity and after testing for X-ray diffraction (XRD), Fourier transform infrared spectroscopy (FTIR) and porosity to N_2_) were prepared for each of the mixtures used in this study (ASTM C192/C192M) [59] which, 24 h after molding, were then demolded and submerged in water until being submitted to testing (28 days of curing) at room temperature in the laboratory.

The test specifications for XRD were: PANalytical X’Pert PRO MPD Alpha1 brand diffractometer, Almelo, The Netherlands) equipped with a Cu Kα_1_ radiation source (λ = 1.5406 Å) X-rays; the test was performed in the range 4° to 80° of 2θ in scanning steps of 2/s. And for the case of FTIR: Alpha-Bruker Fourier Transform Infrared Spectrometer ALPHA-T, with OPUS/Mentor software, WI, USA; the solid samples were prepared by compression with potassium bromide (KBr), grinding the study materials and a portion of KBr with an agate mortar and pestle, the test process included analysis after 32 scans of wave numbers in the 500–4000 cm^−1^ interval, with a resolution of 4 cm^−1^.

### 2.3. Characterization of the Porous Network

Gas (N_2_) adsorption porosimetry was selected as a technique for characterizing the porosity of the RPCs in this study, as it provides sufficient resolution for the porosity in the zones known as micropores and mesopores (less than 2 and 50 nm, respectively) [60]; these zones being where porosity increase is foreseeable due to the presence of mortar adhering to the RA_coarse_. These pores may also be reduced by using the RR.

The total volume of the porosity was determined in order to define pore distribution and obtain the specific surface of the porous materials. This technique is, as a general principle, the quantification of the gas molecules that are found to be attracted to the solid surface of a material, leading to the process known as gas adsorption, in contrast to the phenomena of gas absorption, in which the gas molecules penetrate the solid.

In the case of gas adsorption, the complete test process includes two differential phases, the first of which is known as enrichment (positive adsorption or intrusion) and the second is known as emptying (negative adsorption or desorption). Both curves do not necessarily follow identical trajectories, which leads to different interpretations or explanations of the phenomena that they characterize. The isotherm used in the test establishes both the distribution of pores and the specific surface by means of the Brunauer–Emmett–Teller (BET) method.

The principle governing this technique has been established in previous works [61] in which, using the drop in pressure and volumes (of both the sample and the established test chamber), the ideal gas law is applied to determine the quantity of gas adsorbed, which is related to the total volume of the pores.

For the preparation of the samples studied, and as a requirement of the equipment used (Micromeritics Instrument Corporation brand, TriStar-surface area and porosity analyzer, with TriStar 3000 V6.04 A Software, Norcross, GA, USA), the RPC specimens were manually crushed, selecting the representative particles that satisfy the criteria: size less than 4 mm and total volume up to the maximum allowed in the test (3.3 cm^3^). For each variable studied two identical samples were tested, thereby generating better representation in the study.

## 3. Results

Standard ASTM C642 [58] was used for the characterization of the physical properties to be used in correlation with the porous network of the RPC, obtaining the absorption, apparent density and total water porosity for each sample. Additionally, the ASTM C143/C143M [62] norm was used to determine the workability by means of Abraham’s cone and ASTM C39/C39M [63] to obtain the simple compressive strength of the samples, using a Universal Testing Machine (UTM) (5000 Kg Stepless compression test machine, Wykeham Farrance Ltd., Tring, UK); Table 5 shows the results.

For apparent density and in general terms, the use of the RR produces slight increases (up to 6%) compared to AF-0. Finally, in reference to the porosity of the samples, the highest value was presented by AF-0, with values gradually reducing as the RR factor was increased, until falling to a difference of 1.99% (Comparing AF-7 with control sample), to then increase again in AF-9.

The previous results show, as a possible mechanism of their behavior (sensitive increases in density correlated with RR and inverse correlation with porosity), that the additive is the cause of variations in the matrix of the RPC, given that the rest of the variables in the studied samples have been constant in the tests. For the specific case of open porosity, the RR has a blocking effect [64] on the hardened RPC, which translates into small increases in density.

Regarding the results of simple compression, it can be seen that this increases noticeably as the percentage of RR rises, an effect favored by the densification of the matrix. This effect does not apply to the AF-9 variable, which could be attributed to the saturation of RR in the cementing matrix. With regard to workability, any increase is correlative to the content of the RR, behavior that can be related to the hydrophobic nature of the RR; nevertheless, the values obtained are found to be within an acceptable interval according to the ASTM C192/C192M norm [59].

The dynamic modulus of elasticity (Ed) was also chosen as a mechanical property to be studied in the RPC, as it is one of the properties of resistance that best describes the behavior of concretes, in addition to being easy to obtain and non-destructive (ASTM C215) [65]; the system completed the process via the algorithm and the calculation of the frequencies, correlating mass, density and the propagation of longitudinal elastic waves [66].

Table 6 shows the results obtained, from which it can be seen that the increase in RR content generates increases of 16%–17% for the variables studied compared to the control sample. These results present a direct correlation with density and, consequently, an inverse relationship with the absorption parameter, which is attributed to the reduction in the porosity of concretes. In all cases, the variations in the behavior of the RPCs are related to the different levels of RR content.

**Figure 2:**
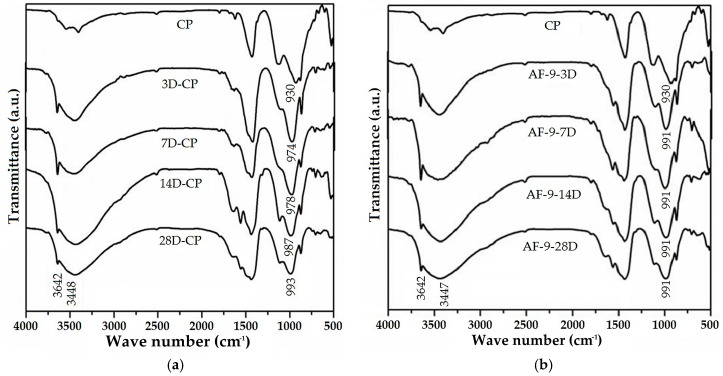
FTIR spectra for ages of 3, 7, 14 and 28 days of hydration: (**a**) Anhydride cement and the cement pastes; (**b**) Anhydride cement and cement pastes with 9% of RR; a.u.: absorbance units.

With the aim of clarifying whether there is any type of evolution in the hydration behavior of the RPC, tests were carried out on pastes with the RR used by means of FTIR. This progress was studied via the identification of three different vibrational bands: (1) the appearance of the Ca(OH)_2_ band, which can be observed around 3640 cm^−1^; (2) the appearance of the H_2_O band, which can be seen around 3437 cm^−1^; and (3) the shift in the C–S–H of the 927 cm^−1^ band (anhydride cement) to 990 cm^−1^ (hydrated cement). Figure 2a shows the progress of hydration for the samples at 3, 7, 14 and 28 days in comparison with the anhydride cement (CP), in which it can be seen that the bands are represented for all samples at 3642 and 3448 cm^−1^, which are associated with water and calcium hydroxide molecules. As all the samples display the characteristic bands of calcium hydroxide and water, the C–S–H band is taken as a reference for establishing the progress of hydration.

In the case of the anhydride cement the C–S–H band is observed around 930 cm^−1^, this position agreeing with previously mentioned studies, while for the samples at 3, 7, 14 and 28 days of hydration the C–S–H band shows displacement of 974 cm^−1^, 978 cm^−1^, 987 cm^−1^ and 993 cm^−1^, which indicates that with more days of hydration the C–S–H band shifts to higher wave numbers, this being due to the advance of hydration caused by the reaction of SiO_4_ during the formation of the C–S–H in the water-cement paste mixtures. It must be emphasized that it had previously been reported that the C–S–H band is to be seen at approximately 991 cm^−1^ in pastes of 28 days hydration [67,68].

Figure 2b shows the FTIR spectra of the anhydride cement and the 9% RR paste at different hydration ages; these spectra show the presence of identical bands, already established in Figure 2a (without RR). The band at 3642 cm^−1^ belongs to the formation of calcium hydroxide and that at 3447 cm^−1^ is due to the vibration of the symmetric tension of the water; 1650 cm^−1^ is attributable to the vibration of the symmetric flexion of the H_2_O contained in the pastes and the 1437 cm^−1^ corresponds to the vibrations of the CO_3_ in the CaCO_3_. That at 1116 cm^−1^ comes from the vibrations of the SO_4_^2−^ of the plaster, while 640 cm^−1^ is due to the asymmetric flexion vibration of the C_3_S silicates; the peaks of 874–711 cm^−1^ are caused by the symmetric and asymmetric flexion vibration of the CO_3_, which moves with time and is a result of the dissolution of the tricalcium silicate.

In contrast to the pastes without RR at 3, 7, 14 and 28 days of hydration (Figure 2a), which show a shift in the C–S–H band of 930–993 cm^−1^ due to the cement hydration, in the pastes with 9% RR at 3, 7, 14 and 28 days (Figure 2b) the C–S–H band remains at 991 cm^−1^, which indicates that optimal hydration is reached after the third day (the same as was observed for the paste without RR at 28 days). In brief, the optimal hydration for the paste without RR is reached at 28 days hydration, whereas the paste with 9% RR achieves it after 3 days. Extrapolating the previous information it can be said that the use of RR in the PMC may accelerate the hydration process (accelerated strength gain), but in any case it cannot lead to or be the cause of the formation of new or different compounds.

**Figure 3:**
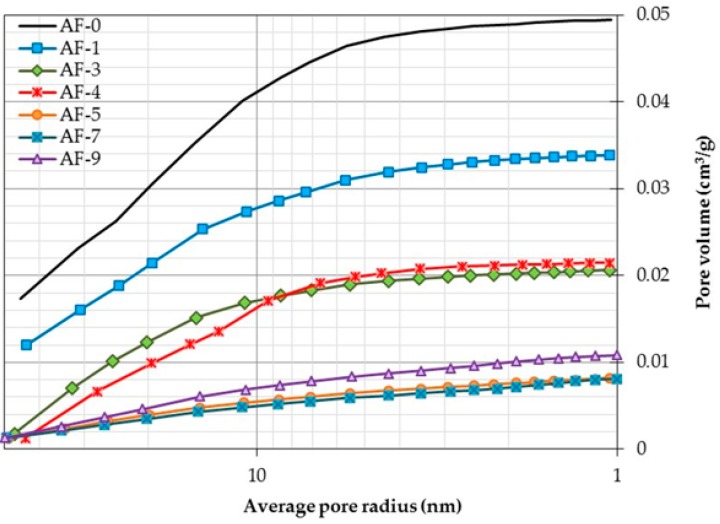
Distribution of pores obtained in the gas adsorption test (intrusion phase) for the samples studied.

The results of the analysis of the N_2_ adsorption tests carried out on the different study samples are presented below (accumulated porosity). Figure 3 details the distribution of the average pore radii at the adsorption stage with all the samples showing average radii, which are mainly found within the mesopore range (1–25 nm), indicating that the possible structural changes in the matrices of these concretes should be studied in the paste [69]. Moreover, it should be noted that depending on the RR content, the porosity of samples AF-1 to AF-7 is reduced when compared to the control sample AF-0. Similarly, AF-9 presents a significant reduction in porosity, although to a lesser extent than the other samples, thus placing it above them (effect of RR overdosage with an inflection point in the mesopores between the samples AF-7 and AF-9, Table 6). As a result, the maximum RR additive content is considered an effect of overdosage, above which the beneficial effects of its use in concrete are less than in those with lower additive contents; in this case the maximum limit is reached when the RR content is greater than 7%.

The RR dosage analysis denotes an inverse correlation between the pore radius reduction and RR dosage, thus causing mechanical improvements similar to those previously presented. This behavior is explained by the formation of what is known as the polymeric film of an RPC, which becomes thicker and more rigid with the increase of RR in the concrete and for which previous modeling exists [70].

In the specific case of AF-9, the trends indicated above do not appear to be valid, as evidenced by the fact that the porosity does not continue to decrease as expected, instead increasing until reaching an increase of 2.8% (compared to AF-5 and AF-7, although still below AF-0). This indicates that a point of RR overdosage exists between content of 7% and 9%, causing a possible non-optimal dispersion of RR within the cementitious matrix, generating zones in which it agglomerates, and making the natural hydration process (continuous porosity) impossible. An RR overdosage increases the packing capacity of cement particles, slowing (or even encapsulating and blocking) the formation of the hydration products responsible for the formation, structure and porosity of the calcium hydroxide (CH) and hydrated calcium silicates (CSH) of a concrete [71], thus leading to a potential cementitious matrix that does not develop due to the blocking of its cement particles, and in this way is prevented from achieving optimal hydration.

**Figure 4:**
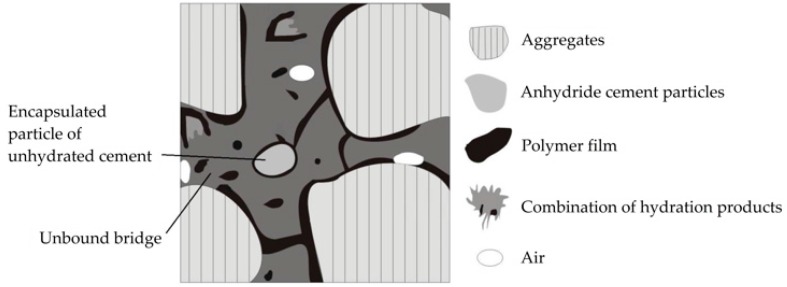
Idealized graphic diagram of microstructural behavior for the proposed new stage of additive overdosage (Stage 4RR_exceeded_).

In the constitutive modeling of RPC, taking that proposed by Beeldens [64] as a model, the four simplified stages are as follows: Stage (1) The formation of the alkaline solution of pores by the initial hydration of the cement; Stage (2) Deposit of RR on the surface of the aggregates with the partial or total coating of some cement particles and, furthermore, the formation of a continuous film with preferential provision on the surface of the hydrates; Stage (3) Continuous hydration and formation of the polymeric film; and Stage (4) The culmination of the final hydration process of the cement, consolidation of the continuous phase of the RR through the cementitious matrix, extraction of the excess water from the pore solution, restriction of the capillary pores, and the formation of bridges between the aggregates. The four stages described are valid for explaining the AF-1, AF-3, AF-4, AF-5 and AF-7 studied here; however, for the AF-9 mixture (overdosage), only the first three stages can be applied, this new case (overdosage) requiring the replacement of Stage 4 by a new stage within the hydration model for an RPC, which has been named Stage 4RR_exceeded_ (see Figure 4). This last stage is also characterized by being the final hydration phase and the consolidation of the continuous film of the RR, but particular partial phases also occur: some of the RR particles lead to agglomerations of isolated products within the matrix, preventing the formation of efficient bridging between the aggregates (unbound bridge). In addition, the anhydride cement particles cannot develop their full crystallization because this is inside the film formed by the RR coverage, thereby preventing the expected densification in the RPC (encapsulated particle).

**Figure 5:**
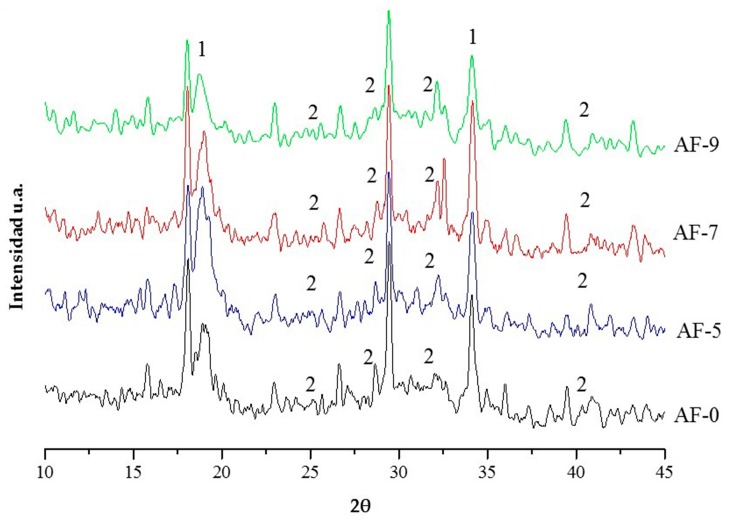
X-ray diffraction (XRD) for the different concrete modified with polymer and recycled aggregate (RPC). 1: calcium hydroxide (CH); 2: hydrated calcium silicates (CSH).

To corroborate the above, the formation of the hydration products was determined for cases AF-5 to AF-7 (see Figure 5) through XRD. Here, the CH (1) peaks can be appreciated, as well as the zones of increase in the formation of CSH (2). In both cases, the behavior described by the diffractograms relates to the detection of more defined and more intense peaks in direct relation to the RR content, taking AF-0 as a reference. However, for case AF-9, the variations in the formation of hydration products show trends towards lower intensity peaks in both hydrates, which could indicate a loss of hydration capacity in the mixture caused by the effect of the RR overdosage (the limitations of the technique itself should be taken into account in this interpretation of the results).

With regard to the quantity of adsorbed gas (total porosity), Table 7 (column 2) shows that the order of the porosity increase in the samples studied is AF-7, AF-5, AF-4, AF-9, AF-3, AF-1 and AF-0 (the order is as expected and similar to that of the other experimental techniques used), reaching a maximum difference of 21.27% between AF-7 with respect to AF-0.

As a constant characteristic of the distribution of radius size (columns 3, 4 and 5, shown as percentage of total porosity), all the samples show higher percentage sizes in the mesoporous zone, reporting a variation in absolute terms of only 3.05, which represents a significance to the order of 5% within these sizes. According to Figure 3, and determining the greater absolute variation of pore size in the other two size zones, the macroporous zone appears to be the key to the variation in the behavior of the mixtures with the use of RR, reporting a 2.64% variation in the absolute value of these sizes. However, compared to the mesoporous zone, this represents a significance of up to 7.33%.

**Figure 6:**
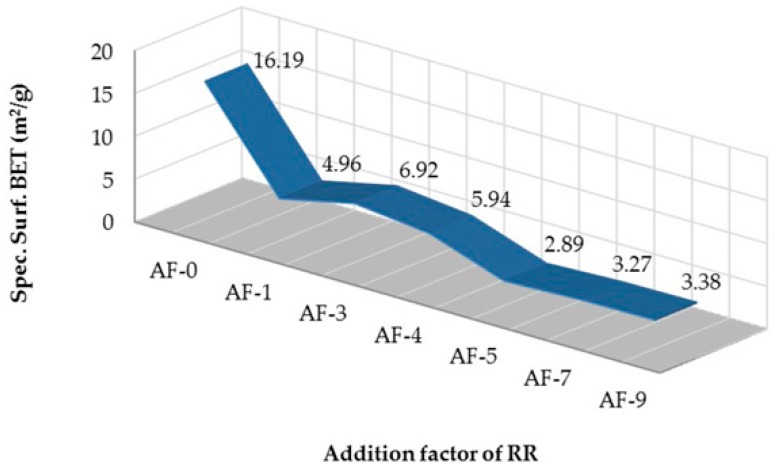
Specific surface determined by means of the Brunauer–Emmett–Teller (BET) method of the mixtures tested.

The behavior of the specific BET surface is presented in Figure 6, which shows a notable decrease for AF-5, AF-7 and AF-9 compared to AF-0, which reaches, in general terms, a value of 71.83% for the group of variables studied. The technological alternatives which are able to provide this type of densification in concretes can only be obtained under strict conditions in the special design of the dosage of the concrete mixes, with a manipulation of the same carried out in situ [72], or finally, by the use of less common components to produce “special concretes” [73].

Within the isothermic curve (theoretical pore radius vs volume of adsorbed N_2_, Figure 3) three radius sizes of interest can be identified, which can be used to describe and understand the behavior of the test samples. These radii are classified into three typologies: that known as the maximum radius, defined as the greatest radius of pore value detected in the test; the medium radius, statistically able to represent the radius of pore value that corresponds to half of the volume of the total adsorption of the test, situating said value on the axis of the abscissa of the graph; and, finally, the critical radius of saturation, which refers to the size of the pore in which gas adsorption initiates a drastic decrease in the adsorbed volume (the end of the test process), and is identified by the change in slope of the curve, becoming a straight line with constant slope. For its determination, the first pore radius causing a change in the slope of the curve with an angle greater than 5° was chosen, taking 87.1°–86.5° as a common range from all the samples for its possible identification, measured from the vertical axis as origin and with reference to the horizontal axis in an anticlockwise direction.

**Figure 7:**
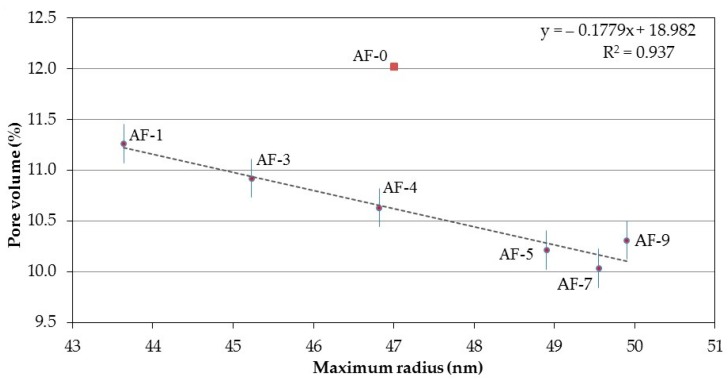
Correlation between the Maximum radius and the Pore volume (to water) of the different concretes modified with polymer and recycled aggregate (RPC).

For the first of the radii of interest (maximum radius), a correlation between this and the physical properties of the RPC was established, indicating that the porosity (to water) reaches a high determination factor (see Figure 7) for the adjustment of a straight line with a constant inverse slope (an increase in the maximum pore radius produces lower pore volumes). Obtaining the best fit through a linear type equation could show a high and direct correlation between both parameters and therefore help to explain or understand the porous network of the RPC. However, a priori, it can be said that it appears that the change from lower radius maximum pores to those with a larger radius reduces total porosity to water. This could be explained by the hypothesis that while RR causes the porosity of average or small radii to be sealed, it is unable to do this with large radius pores, which in turn remain open and are able to contain a higher quantity of water absorbed in the RPC.

**Figure 8:**
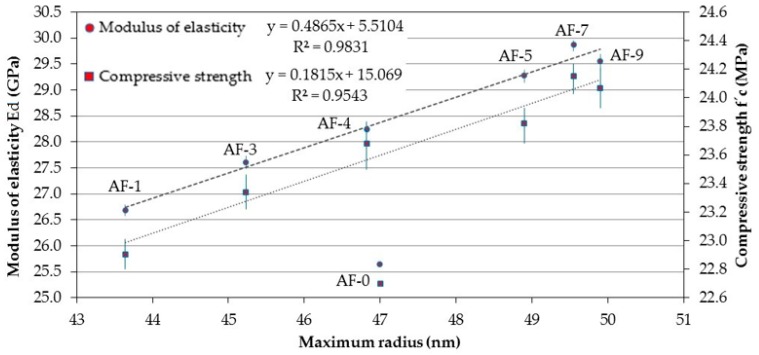
Correlation between the Maximum radius and, the Modulus of elasticity and Compressive strength, of the different concretes modified with polymer and recycled aggregate (RPC).

In terms of the correlation indicated in Figure 8 for the maximum radius, Ed and compressive strength, the best determination factor attained was R^2^ = 0.9831 for Ed and R^2^ = 0.9543 for compressive strength, with a type of linear curve fit. However, in contrast to the previous case, on this occasion there is an ascending incremental relationship. The explanatory hypothesis for this is that the technique used in the determination of Ed is based on the measurement of the frequency of impact through the element, observing that when the RR causes the change in the maximum radius (small or medium) to increase, the remaining porosity of the element is sealed or densified. Therefore, this generates more compact and continuous elements that facilitate the improved passage and reception of the signal and, thus, higher Ed values. Regarding the compressive strength, the behavior is understood as being the same as in the usual concretes; that is, a concrete matrix is more resistant as it becomes denser.

**Figure 9:**
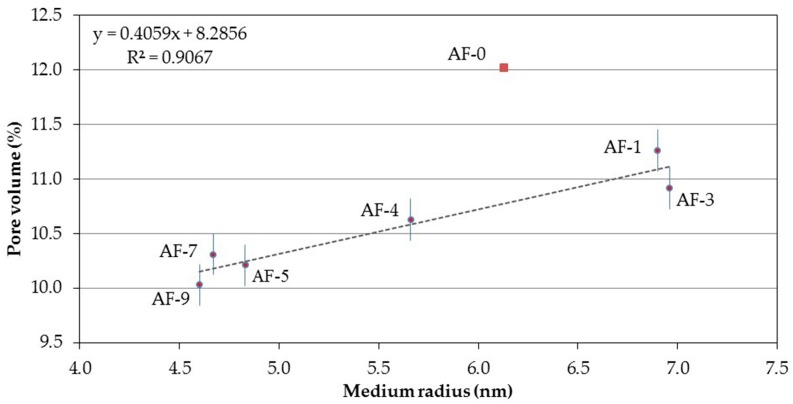
Correlation between the Medium radius and the Pore volume of the different concretes modified with polymer and recycled aggregate (RPC).

Relating the medium radius and total porosity (to water) obtains a straight line of fit with an ascending slope, which in this case (due to its type of fit and direction) is one of the correlations between the porous network and physical properties of a concrete that can be better explained (see Figure 9). This is because the medium radius is a representative value of the entire porosity of a concrete; increases in porosity to water are the natural result of medium radius increases.

**Figure 10:**
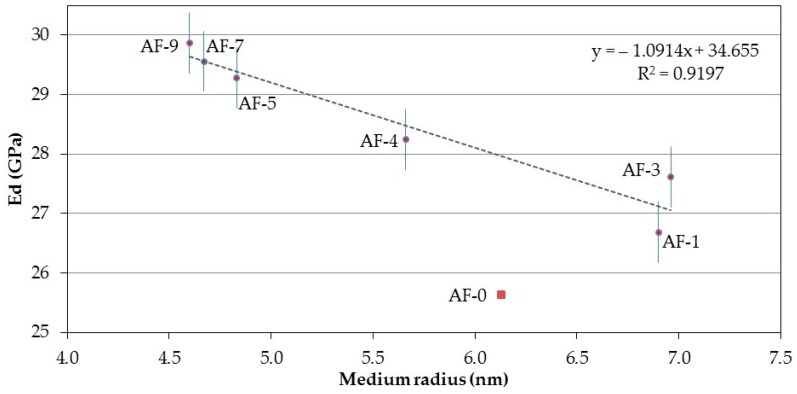
Correlation between the Medium radius and the Modulus of elasticity of the different concretes modified with polymer and recycled aggregate (RPC).

With regard to the medium radius, it is possible to establish a correlation between this and the corresponding Ed (Figure 10) using a linear regression type with an inverse slope. The linear nature of the equation denotes a strong link between the calibration parameter (medium radius) and the independent variable Ed. This property is therefore better explained for the RPC (with the medium radius) than with the maximum radius. This reasoning could be due to the origin or nature of the medium radius parameter (pore that is the equivalent to half of the volume of porosity recorded in the adsorption of N_2_ test).

**Figure 11:**
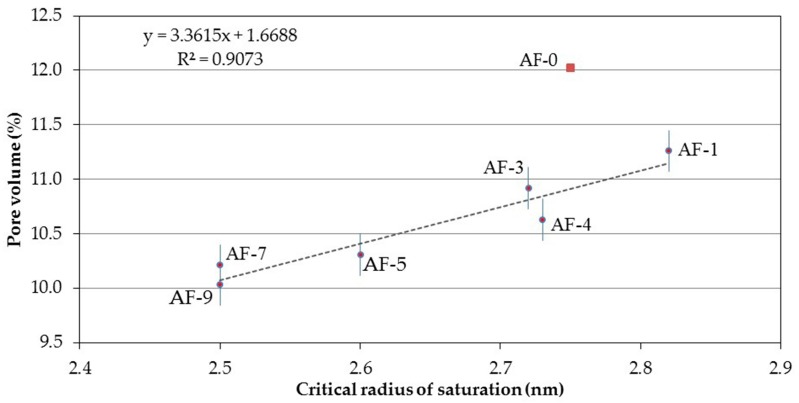
Correlation between the Critical radius of saturation and the Pore volume of the different concretes modified with polymer and recycled aggregate (RPC).

Using what was defined as the critical radius of saturation and correlated with Ed (Figure 11), a linear fit with an ascending slope (similar for the case with the medium radius and pore volume) is obtained. Taking into account the fact that the slope in this case is more pronounced than that in the medium radius (implying faster changes or an accelerated variation of correlation), the interpretation of this term, in similar situations, should be selected as that of most interest as a parameter allowing the mechanical phenomenon of the Ed of the RPC (greater possible path of the line) to be explained or defined more adequately.

**Figure 12:**
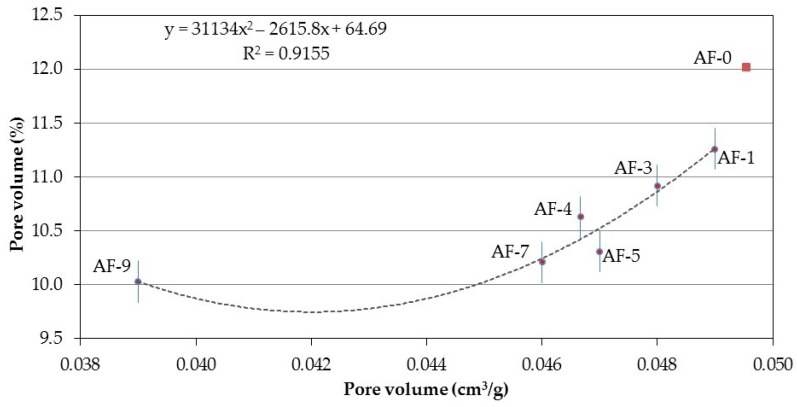
Correlation between the Pore volume (N_2_) and the Pore volume (to water) of the different concretes modified with polymer and recycled aggregate (RPC).

The relationship between the porosity obtained via the adsorption of N_2_ and the total porosity to water (see Figure 12) proved highly significant in this research, which validates both techniques as comparable in terms of their capacity to characterize the porous structures of the RPC. On the other hand, of the previously presented different correlations that try to explain the porosity to water of the PRC, this one is of quadratic polynomial type, the interpretation being that it is necessary to consider some other variable for the explanation of the phenomenon (not established in this research).

**Figure 13:**
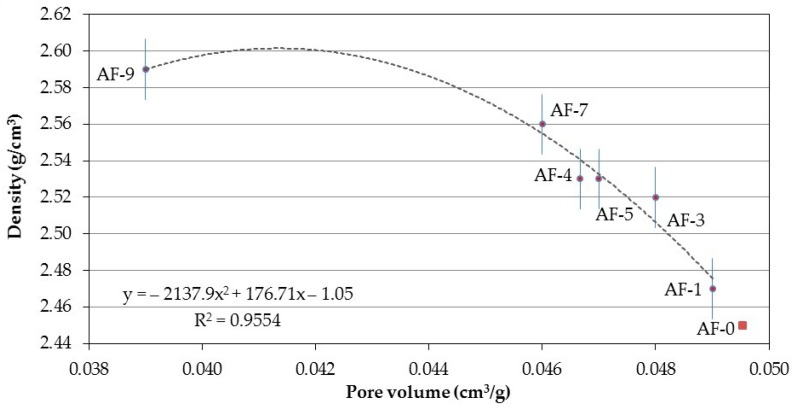
Correlation between the Pore volume and the Density of the different concretes modified with polymer and recycled aggregate (RPC).

Relating the N_2_ pore volume to the density (see Figure 13), an equation of the decreasing polynomial type obtains the best fit, which corroborates that the reduction in total porosity improves the density results although with a tendency to increase this parameter due to the nature of the test itself.

## 4. Conclusions

The study of the physical, mechanical and microstructural properties of the RPCs has permitted their behavior to be described, which shows improvements when RR additive percentages of between 1% and 9% are used in their matrix. Among the different properties studied, the porosity of the resulting matrix becomes one of the most favorable microstructural improvements obtained, achieving reductions of up to 16.55% less than the control specimen, while for water absorption values of up to 22% less were obtained. For the case of the dynamic modulus of elasticity, it is possible to obtain improvements of 16.50%.

Considering the constitutive model of the RPC behavior from previous studies, the reduction in the porosity of the RPC is attributed to the formation of a cementitious matrix impregnated by a polymeric network, which is constituted among the bridging bounds of the RR particles. These bridging bounds are positioned with preferential orientation in order to bond with the aggregates, thus leading to the physical obstruction of the continuous porosity used for the conduction of fluids.

It is verified that RC considered as porous can mitigate this problem if they are designed using the RR additive, thereby allowing the use of RA_coarse_ to be equivalent to NA_coarse_ and thus achieving environmental improvements.

A change in the favorable behavior trend of the RPC has been established when the RR contents are between 7% and 9%, which leads to a reversal of the improvements attained with the previous dosage. Nevertheless, for the 9% RR content studied here, the properties of these variables present improvements when compared to the control sample (although fewer than those recorded for lower RR content).

This behavior caused by overdosage is due to the agglomeration of RR particles that are not completely linked, obstructing the pore sealing bridges, which thus remain isolated while also coating the cement hydrates and the particles of the aggregates that are encapsulated (loss of the reactive capacity for hydration contrasted by XRD). This new stage was designated Stage 4RR_exceeded_, which describes a new alternative for the behavior of overdosage in the use of RR.

The total porosity to water and the porosity by adsorption of N_2_ are techniques that reliably express the correlations between the results and the other properties describing the behavior of the RPC mixtures studied. Among these correlations this study has also verified, through ascending linear correlation, the best affinity with the results obtained. Finally, the parameter for the medium radius (obtained for the adsorption of N_2_) enables the precise description and correlation established between the porous network of the RPC studied here, as well as the properties for Ed and the pore volume (to water), thus allowing their behavior to be explained in part.

## Figures and Tables

**Table 1:** Properties of the original concrete (OC) used as recycled aggregates.

Property	Value
Absorption (%)	6.91
Density (g/cm^3^)	2.36
Porosity (%)	17.87
Rebound index	28.15

**Table 2:** Properties of recycled aggregates (RA_coarse_) and natural (NA_coarse_ and NA_fine_) used in the experiment.

Property	RA_coarse_	NA_coarse_	NA_fine_
Absorption (%)	6.27	1.19	1.88
Density (g/cm^3^)	2.38	2.56	2.59
Fineness modulus	-	-	2.53
Maximum size (mm)	19.05	19.05	4.76

**Table 3:** Experimental techniques.

Tests/Technical	Normative
Workability	ASTM C143/143M
Apparent density	ASTM C642
Pore volume	
Compressive strength	ASTM C39/C39M
Dynamic modulus of elasticity	ASTM C215
X-ray diffraction (XRD)	-
Fourier transform infrared spectroscopy (FTIR)	-
Gas (N_2_) adsorption porosimetry	-

**Table 4:** Quantities of materials used in the dosage of experimental mixtures.

Component	Classification of Mixtures ^1^						
	AF-0	AF-1	AF-3	AF-4	AF-5	AF-7	AF-9
Cement (Kg/m^3^)	350.00						
Water (Kg/m^3^)	175.00						
RA_coarse_ (Kg) (4.76–12.70 mm)	262.50						
NA_coarse_ (Kg)(4.76–12.70 mm)	787.50						
NA_fine_ (Kg) (1.00–4.76 mm)	700.00						
RR (Kg)	-	3.50	10.50	14.00	17.50	24.50	31.50
w/c	0.5						

^1^ AF = Addition Factor of RR, the second term showing the content thereof in the mixture.

**Table 5:** Physical properties of concrete modified with polymer and recycled aggregate (RPC).

Parameters	Classification of Mixtures						
	AF-0	AF-1	AF-3	AF-4	AF-5	AF-7	AF-9
Apparent density (g/cm^3^)	2.45	2.47	2.52	2.53	2.56	2.59	2.53
Pore volume (%)	12.02	11.26	10.92	10.63	10.21	10.03	10.31
Compressive strength (MPa)	22.70	22.90	23.34	23.86	23.82	24.15	24.07
Workability (cm)	8.55	8.50	8.50	8.45	8.50	8.65	8.95

**Table 6:** Dynamic modulus of elasticity (Ed).

Mixture	Ed (GPa)
AF-0	25.64
AF-1	26.68
AF-3	27.61
AF-4	28.24
AF-5	29.28
AF-7	29.87
AF-9	29.56

**Table 7:** Distribution of the pore size ranges obtained from the adsorbed gas test.

Mixture	Total Porosity (cm^3^/g)	Macropores ^1^ (%)	Mesopores ^2^ (%)	Micropores ^3^ (%)
AF-0	0.04954	38.34	59.62	2.04
AF-1	0.04900	33.38	64.94	1.68
AF-3	0.04800	38.34	59.62	2.04
AF-4	0.04667	34.03	63.39	2.58
AF-5	0.04600	32.44	65.21	2.35
AF-7	0.03900	38.51	59.51	1.98
AF-9	0.04700	39.51	58.37	2.12

^1^ Size > 25 nm; ^2^ Size 1–25 nm; ^3^ Size < 1 nm.
